# Briarenolides K and L, New Anti-Inflammatory Briarane Diterpenoids from an Octocoral *Briareum* sp. (Briareidae)

**DOI:** 10.3390/md13021037

**Published:** 2015-02-13

**Authors:** Yin-Di Su, Tzu-Rong Su, Zhi-Hong Wen, Tsong-Long Hwang, Lee-Shing Fang, Jih-Jung Chen, Yang-Chang Wu, Jyh-Horng Sheu, Ping-Jyun Sung

**Affiliations:** 1 Department of Marine Biotechnology and Resources, Asia-Pacific Ocean Research Center, National Sun Yat-sen University, Kaohsiung 804, Taiwan; E-Mails: gobetter04@yahoo.com.tw (Y.-D.S.); wzh@mail.nsysu.edu.tw (Z.-H.W.); 2 National Museum of Marine Biology and Aquarium, Pingtung 944, Taiwan; 3 Department of Beauty Science, Meiho University, Pingtung 912, Taiwan; E-Mail: a081002@mail.tsmh.org.tw; 4 Antai Medical Care Cooperation Antai Tian-Sheng Memorial Hospital, Pingtung 928, Taiwan; 5 Graduate Institute of Natural Products, Chang Gung University, Taoyuan 333, Taiwan; E-Mail: htl@mail.cgu.edu.tw; 6 Department of Sport, Health and Leisure, Cheng Shiu University, Kaohsiung 833, Taiwan; E-Mail: lsfang@csu.edu.tw; 7 Department of Pharmacy, Tajen University, Pingtung 907, Taiwan; E-Mail: jjchen@tajen.edu.tw; 8 School of Pharmacy, College of Pharmacy, China Medical University, Taichung 404, Taiwan; E-Mail: yachwu@mail.cmu.edu.tw; 9 Chinese Medicine Research and Development Center, China Medical University Hospital, Taichung 404, Taiwan; 10 Graduate Institute of Natural Products, Kaohsiung Medical University, Kaohsiung 807, Taiwan; 11 Center for Molecular Medicine, China Medical University Hospital, Taichung 404, Taiwan; 12 Graduate Institute of Marine Biology, National Dong Hwa University, Pingtung 944, Taiwan

**Keywords:** *Briareum*, briarane, octocoral, anti-inflammatory, iNOS

## Abstract

Two new briarane-type diterpenoids, briarenolides K (**1**) and L (**2**), were isolated from an octocoral identified as *Briareum* sp. The structures of new briaranes **1** and **2** were elucidated by spectroscopic methods. In the* in vitro* anti-inflammatory effects test, briaranes **1** and **2** were found to significantly inhibit the accumulation of the pro-inflammatory iNOS protein of the lipopolysaccharide (LPS)-stimulated RAW264.7 macrophage cells.

## 1. Introduction

**Scheme 1:**
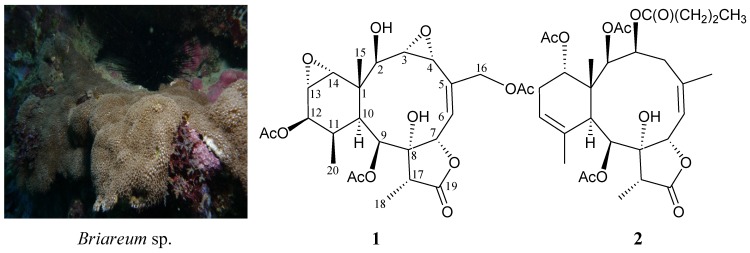
The octocoral *Briareum* sp. and the structures of briarenolides K (**1**) and L (**2**).

Among the diterpenoids isolated from octocorals, the 3,8-cyclized cembranoidal diterpenes (briarane) are major representative compounds. Briarane-type natural products are found only in marine organisms and mainly from octocorals [1,2,3,4,5]. The compounds of this type are suggested to be originally synthesized by host corals [6,7] and have been proven to possess various interesting bioactivities [1,2,3,4,5]. The octocorals belonging to the genus *Briareum* (phylum Cnidaria, class Anthozoa, subclass Octocorallia, order Gorgonacea, suborder Scleraxonia, family Briareidae) have been proven to be the most important source of briarane analogues [1,2,3,4,5]. The genus *Briareum* is distributed widely in the Indo-Pacific Ocean and the Caribbean Sea and is placed taxonomically within the orders Gorgonacea and Alcyonacea [8,9,10,11]. Previous chemical investigations of octocorals belonging to the genus *Briareum*, collected off the waters of Taiwan, at the intersection of the Kuroshio current and the South China Sea surface current, have yielded numbers of briarane analogues [12,13,14,15,16,17,18,19,20,21,22,23,24,25,26,27,28,29,30,31,32,33,34,35,36,37,38,39,40,41,42,43,44]. In our continuing studies of this interesting organism, a sample collected at the Southern Tip, Taiwan, identified as *Briareum* sp. [45,46,47,48,49], yielded two new briaranes, briarenolides K (**1**) (Scheme 1 and Supplementary Figures S1–S7) and L (**2**) (Scheme 1 and Supplementary Figures S8–S14). In this paper, we report the isolation, structure determination and anti-inflammatory activity of briaranes **1** and **2**.

## 2. Results and Discussion

Briarenolide K (**1**), ${\left[\alpha \right]}_{D}^{27}$ −291 (*c* 0.2, CHCl_3_), was isolated as a white powder. The molecular formula of **1** was established as C_26_H_34_O_12_ (ten degrees of unsaturation) from a sodium adduct at *m/z* 561 in the electrospray ionization mass spectrum (ESIMS) and further supported by the high-resolution electrospray ionization mass spectrum (HRESIMS) at *m/z* 561.19435 (calcd. for C_26_H_34_O_12_ + Na, 561.19425). The IR spectrum of **1** showed bands at 3366, 1781 and 1733 cm^−1^, consistent with the presence of hydroxy, γ-lactone and ester carbonyl groups.

**Table 1:** ^1^H (400 MHz, CDCl_3_) and ^13^C (100 MHz, CDCl_3_) NMR data and ^1^H–^1^H COSY and HMBC correlations for briarane **1**.

Position	δ_H_ (*J* in Hz)	δ_C_, Multiple	^1^H–^1^H COSY	HMBC
1		39.2, C		
2	3.12 br d (9.6)	75.9, CH	H-3, OH-2	n. o. ^a^
3	3.31 dd (9.6, 4.0)	60.3, CH	H-2, H-4	C-1
4	4.09 br s	58.2, CH	H-3, H-6	n .o.
5		136.3, C		
6	5.81 dddd (9.2, 1.2, 0.8, 0.8)	122.9, CH	H-4, H-7, H_2_-16	C-16
7	5.32 d (9.2)	76.6, CH	H-6	C-5, -8
8		81.8, C		
9	5.35 d (8.4)	69.3, CH	H-10	C-8, -17, acetate carbonyl
10	1.82 dd (8.4, 2.4)	36.8, CH	H-9, H-11	C-1, -2, -8, -9, -11, -15, -20
11	2.17 m	36.5, CH	H-10, H-12, H_3_-20	n. o.
12	4.94 d (4.8)	71.7, CH	H-11, H-13	C-10, -11, -13, -14, -20,
				acetate carbonyl
13	3.27 d (4.0)	58.1, CH	H-12, H-14	C-1
14	3.34 d (4.0)	62.7, CH	H-13	C-1, -10
15	1.20 s	15.2, CH_3_		C-1, -2, -10, -14
16a	4.67 d (15.6)	64.6, CH_2_	H-6, H-16b	C-5, -6
b	4.80 d (15.6)		H-6, H-16a	C-5, -6
17	2.39 q (6.8)	43.7, CH	H_3_-18	C-18, -19
18	1.21 d (6.8)	6.3, CH_3_	H-17	C-8, -17, -19
19		175.6, C		
20	1.08 d (6.8)	9.7, CH_3_	H-11	C-10, -11, -12
9-OAc		169.5, C		
	2.20 s	21.8, CH_3_		Acetate carbonyl
12-OAc		170.3, C		
	2.11 s	21.0, CH_3_		Acetate carbonyl
16-OAc		172.2, C		
	2.19 s	21.2, CH_3_		Acetate carbonyl
OH-2	3.55 br s		H-2	n. o.
OH-8	3.73 br s			n. o.

^a^ n. o. = not observed.

The ^13^C NMR and distortionless enhancement polarization transfer (DEPT) spectral data showed that this compound has 26 carbons (Table 1), including six methyls, an sp^3^ oxymethylene, eleven sp^3^ methines (including eight oxymethines), two sp^3^ quaternary carbons (including an oxygenated quaternary carbon), an sp^2^ methine and five sp^2^ quaternary carbons (including four carbonyls). From ^1^H and ^13^C NMR spectra (Table 1), **1** was found to possess three acetoxy groups (δ_H_ 2.20, 2.19, 2.11, each 3H × s; δ_C_ 169.5, 172.2, 170.3, 3 × C; 21.8, 21.2, 21.0, 3 × CH_3_), a γ-lactone moiety (δ_C_ 175.6, C-19) and a trisubstituted olefin (δ_H_ 5.81, 1H, dddd, *J* = 9.2, 1.2, 0.8, 0.8 Hz, H-6; δ_C_ 136.3, C-5; 122.9, CH-6). The presence of two disubstituted epoxy groups was established from the signals of four oxymethines at δ_C_ 60.3 (CH-3), 58.2 (CH-4), 58.1 (CH-13) and 62.7 (CH-14) and further confirmed by the proton signals at δ_H_ 3.31 (1H, dd, *J* = 9.6, 4.0 Hz, H-3), 4.09 (1H, br s, H-4), 3.27 (1H, d, *J* = 4.0 Hz, H-13) and 3.34 (1H, d, *J* = 4.0 Hz, H-14). On the basis of the above unsaturation data, **1** was concluded to be a diterpenoid molecule possessing five rings.

From the ^1^H–^1^H correlation spectroscopy (COSY) spectrum of **1** (Table 1), it was possible to establish the separate system that maps out the proton sequences from H-2/H-3/H-4, H-4/H-6 (by allylic coupling), H-6/H-7 and H-9/H-10. These data, together with the heteronuclear multiple-bond coherence (HMBC) correlations between H-3/C-1, H-7/C-5, -8, H-9/C-8 and H-10/C-1, -2, -8, -9, established the connectivity from C-1 to C-10 in the ten-membered ring (Table 1). The methylcyclohexane ring, which is fused to the ten-membered ring at C-1 and C-10, was elucidated by the ^1^H–^1^H COSY correlations between H-10/H-11/H-12/H-13/H-14 and H-11/H_3_-20 and by the HMBC correlations between H-10/C-11, -20, H-12/C-10, -11, -13, -14, -20, H-13/C-1, H-14/C-1, -10 and H_3_-20/C-10, -11, -12. The ring junction C-15 methyl group was positioned at C-1 from the HMBC correlations between H_3_-15/C-1, -2, -10, -14 and H-10/C-15. The acetate esters at C-9 and C-12 were established by the correlations between H-9 (δ_H_ 5.35), H-12 (δ_H_ 4.94) and the acetate carbonyls at δ_C_ 169.5 and 170.3, respectively, in the HMBC spectrum of **1**. The methyl acetate group at C-5 was confirmed by the HMBC correlations between the oxygen-bearing methylene protons at δ_H_ 4.67 and 4.80 (H-16a/b) and the olefinic carbons C-5 and C-6; and further confirmed by the allylic couplings between H_2_-16 and H-6, although there is no HMBC correlation found between H_2_-16 and the acetate carbonyl at δ_C_ 172.2. The presence of a hydroxy group at C-2 was deduced from the ^1^H–^1^H COSY correlation between a hydroxy proton (δ_H_ 3.55) and H-2 (δ_H_ 3.12). Thus, the remaining hydroxy group is positioned at C-8 and an oxygen-bearing quaternary carbon at δ_C_ 81.8. These data, together with the ^1^H–^1^H COSY correlation between H-17 and H_3_-18 and the HMBC correlations between H-9/C-17, H-17/C-18, -19 and H_3_-18/C-8, -17, -19, were used to establish the molecular framework of **1**.

**Figure 1:**
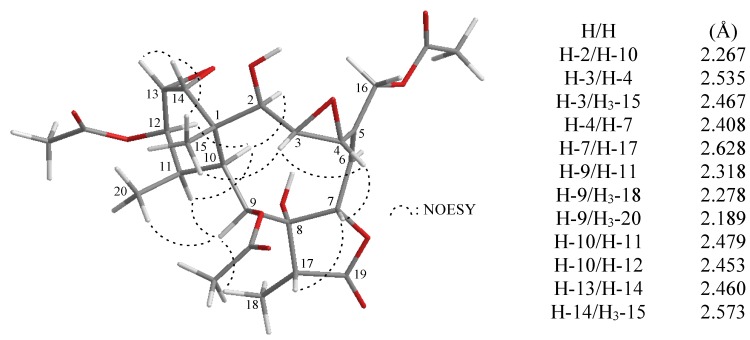
The computer-generated model of **1** using MM2 force field calculations and the calculated distances (Å) between selected protons with key NOESY correlations.

In all naturally-occurring briarane-type natural products, H-10 is *trans* to the C-15 methyl group at C-1, and these two groups are assigned as α- and β-oriented in most briarane derivatives [1,2,3,4,5]. The relative configuration of **1** was elucidated from the interactions observed in a nuclear Overhauser effect spectroscopy (NOESY) experiment and was found to be compatible with that of **1** offered by computer modeling (Figure 1) [50] and that obtained from vicinal proton coupling constant analysis. In the NOESY experiment of **1**, the correlations of H-10 with H-2, H-11 and H-12, but not with H_3_-15 and H_3_-20, indicated that H-2, H-10, H-11 and H-12 were situated on the same face and were assigned as α protons, since the Me-15 and Me-20 are β-substituents at C-1 and C-11, respectively. H-14 showed correlations with H-13 and Me-15, but not with H-10, as well as a lack of coupling was detected between H-12 and H-13, indicating that the dihedral angle between H-12 and H-13 is approximately 90° and the 13,14-epoxy group has an α-orientation [51]. H-9 was found to show responses to H-11, H_3_-18 and H_3_-20. From modeling analysis, H-9 was found to be close H-11, H_3_-18 and H_3_-20 when H-9 was α-oriented. H-3 correlated with Me-15 and H-4, but not with H-2 and H-10, and a large coupling constant (*J* = 9.6 Hz) was detected between H-2 and H-3, indicating that the 3,4-epoxy group was α-oriented. In addition, H-4 showed a correlation with H-7, and a large coupling constant (*J* = 9.2 Hz) was detected between H-6 and H-7, indicating that the dihedral angle between H-6 and H-7 is approximately 180°, and H-7 was β-oriented [51]. Furthermore, H-7 exhibited a correlation with H-17, but not with the C-8 hydroxy proton, suggesting that the H-17 and 8-hydroxy group were β- and α-oriented in the γ-lactone moiety, respectively, by modeling analysis. Based on the above findings, the structure of **1** was established unambiguously.

Briarenolide L (**2**) had a molecular formula of C_30_H_42_O_11_ as deduced from HRESIMS at *m/z* 601.26221 (C_30_H_42_O_11_ + Na, calcd. 601.26193). Thus, ten degrees of unsaturation were determined for **2**. The IR spectrum showed bands at 3461, 1780 and 1739 cm^−1^, consistent with the presence of hydroxy, γ-lactone and ester carbonyl groups in the structure of **2**. From the ^13^C spectral data of **2** (Table 2), two trisubstituted olefins were deduced from the signals of four carbons at δ_C_ 143.2 (C-5), 121.2 (CH-6), 131.6 (C-11) and 122.2 (CH-12). In the ^13^C NMR spectrum of **2**, five carbonyl resonances at δ_C_ 176.1, 172.3, 170.3, 169.8 and 169.2 (5 × C) confirmed the presence of a γ-lactone and four other ester groups. In the ^1^H NMR spectrum of **2** (Table 2), an *n*-butyrate group (δ_H_ 0.94, 3H, t, *J* = 7.2 Hz; 1.60, 2H, m; 2.21, 2H, t, *J* = 7.6 Hz) and three acetyl methyls (δ_H_ 2.24, 2.10, 1.98, each 3H × s) were observed. Based on the above data, metabolite **2** was found to be a tricyclic compound.

^1^H–^1^H couplings in the COSY spectrum of **2** enabled identification of the C-2/-3/-4, C-6/-7, C-9/-10, C-12/-13/-14, C-6/-16 (by allylic coupling), C-12/C-20 (by allylic coupling) and C-17/-18 units (Table 2), which were assembled with the assistance of an HMBC experiment. The HMBC correlations between the protons and carbons of **2**, such as H-4α/C-5, H-4β/C-3, H-6/C-4, -5, -8, H-7/C-6, H-9/C-1, -7, -8, -10, -11, -17, H-10/C-8, -9, -11, -12, -14, H-13α/C-12, H-14/C-2, H-17/C-8, -18, -19 and H_3_-18/C-8, -17, -19, permitted the elucidation of the main carbon skeleton of **2** (Table 2). The vinyl methyls at C-5 and C-11 were confirmed by the HMBC correlations between H_3_-16/C-4, -5, -6 and H_3_-20/C-10, -11, -12 and further supported by the allylic couplings between H-6/H_3_-16 and H-12/H_3_-20, respectively. The C-15 methyl group was positioned at C-1 from the HMBC correlations between H_3_-15/C-1, -2, -10, -14. In addition, the carbon signal at δ_C_ 172.3 (C) was correlated with the signal of the methylene protons at δ_H_ 2.21 and 1.60 in the HMBC spectrum and was consequently assigned as the carbon atom of the *n*-butyrate carbonyl. The acetate esters at C-2 and C-9 were established by the correlations between H-2 (δ_H_ 5.51), H-9 (δ_H_ 5.34) and the acetate carbonyls at δ_C_ 169.2 and 169.8, respectively, observed in the HMBC spectrum of **2**. Due to the absence of HMBC correlations for H-3 (δ_H_ 4.76) and H-14 (δ_H_ 4.92) and the ester carbonyls, the positions for the remaining acetoxy and *n*-butyroxy groups could not be determined by this method.

**Table 2:** ^1^H (400 MHz, CDCl_3_) and ^13^C (100 MHz, CDCl_3_) NMR data and ^1^H–^1^H COSY and HMBC correlations for briarane **2**.

Position	δ_H_ (*J* in Hz)	δ_C_, Multiple	^1^H–^1^H COSY	HMBC
1		42.1, C		
2	5.51 d (3.6)	72.5, CH	H-3	Acetate carbonyl
3	4.76 ddd (3.6, 3.2, 3.2)	72.3, CH	H-2, H_2_-4	n. o.
4α	2.13 m	33.7, CH_2_	H-3, H-4β	C-5
β	3.31 dd (16.0, 3.2)		H-3, H-4α	C-3
5		143.2, C		
6	5.53 s	121.2, CH	H-7, H_3_-16	C-4, -5, -8, -16
7	5.53 s	79.8, CH	H-6	C-6
8		84.6, C		
9	5.34 d (1.2)	77.2, CH	H-10	C-1, -7, -8, -10, -11, -17,
				acetate carbonyl
10	2.96 br s	43.8, CH	H-9	C-8, -9, -11, -12, -14
11		131.6, C		
12	5.49 br s	122.2, CH	H_2_-13, H_3_-20	n. o.
13α	2.17 m	28.2, CH_2_	H-12, H-13β, H-14	C-12
β	2.35 m		H-12, H-13α, H-14	n. o.
14	4.92 dd (10.0, 7.2)	73.5, CH	H_2_-13	C-2
15	1.59 s	17.3, CH_3_		C-1, -2, -10, -14
16	1.85 br s	23.9, CH_3_	H-6	C-4, -5, -6
17	2.91 q (7.2)	42.1, CH	H_3_-18	C-8, -18, -19
18	1.11 d (7.2)	7.4, CH_3_	H-17	C-8, -17, -19
19		176.1, C		
20	1.79 d (1.2)	22.2, CH_3_	H-12	C-10, -11, -12
2-OAc		169.2, C		
	2.10 s	21.0, CH_3_		Acetate carbonyl
9-OAc		169.8, C		
	2.24 s	21.0, CH_3_		Acetate carbonyl
14-OAc		170.3, C		
	1.98 s	21.0, CH_3_		Acetate carbonyl
3-OC(O)CH_2_CH_2_CH_3_		172.3, C		
1′ 2′ 3′ 4′	2.21 t (7.6)	35.9, CH_2_	H_2_-3′	C-1′, -3′, -4′
	1.60 m	18.0, CH_2_	H_2_-2′, H_3_-4′	C-1′, -2′, -4′
	0.94 t (7.2)	13.6, CH_3_	H_2_-3′	C-2′, -3′

^a^ n. o. = not observed.

**Figure 2:**
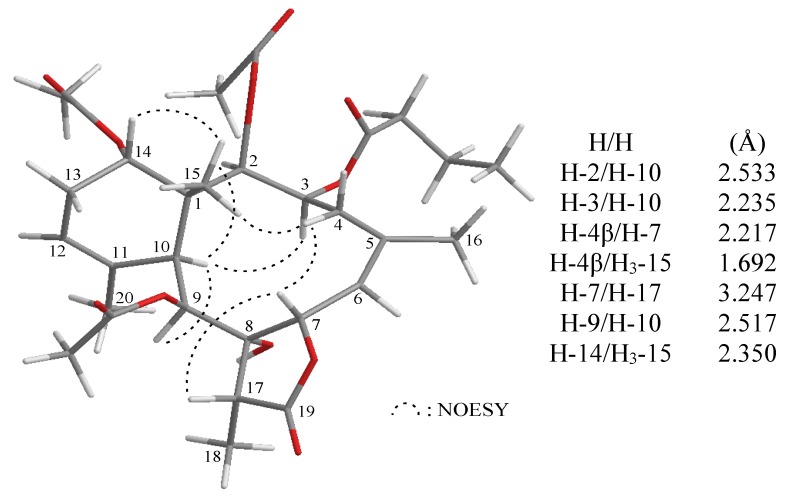
The computer-generated model of **2** using MM2 force field calculations and the calculated distances (Å) between selected protons with key NOESY correlations.

NOESY measurements were carried out in order to deduce the relative stereochemical features of **2** (Figure 2). Thus, H_3_-15 gave a correlation with H-14, but not with H-10, indicating that Me-15 and H-14 are located on the same face (assigned as the β-face) and that H-10 lies on the opposite side, the α-face. It was found that one of the methylene protons at C-4 (δ_H_ 3.31) exhibited correlations with H_3_-15 and H-7, but not with H-3, and therefore, it was assigned as H-4β and the other C-4 proton (δ_H_ 2.13) as H-4α. H-10 showed correlations with H-2, H-3 and H-9, indicating that the ester groups at C-2, C-3 and C-9 were β-oriented. Moreover, H-7 exhibited a correlation with H-17, supporting that H-17 and the hydroxy group at C-8 were positioned on the β- and α-positions at C-17 and C-8 in the γ-lactone moiety of **2** by modeling analysis. Fortunately, the methylene protons of the *n*-butyrate group at δ_H_ 2.21 exhibited correlation with H-2, indicating that the *n*-butyrate group should be positioned at C-3, and the remaining acetoxy group is positioned at C-14, an oxymethine at δ_C_ 73.5.

**Figure 3:**
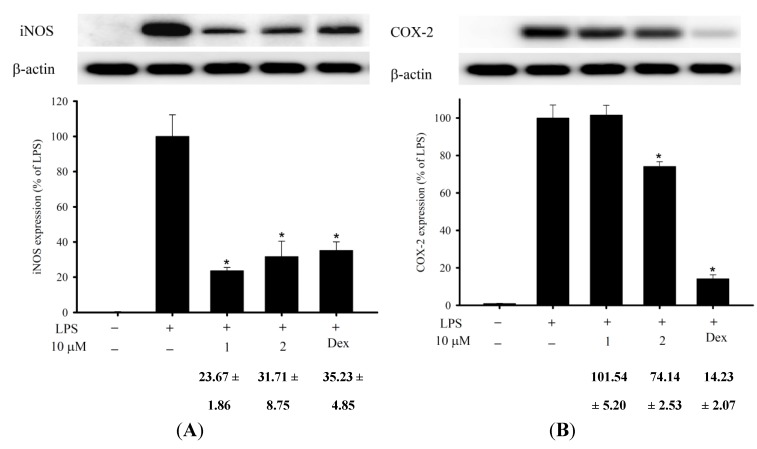
Effects of Compounds **1 **and **2 **on pro-inflammatory iNOS and COX-2 protein expression in the LPS-stimulated murine macrophage cell line, RAW264.7. (**A**) Relative density of iNOS immunoblot; (**B**) relative density of COX-2 immunoblot. The relative intensity of the LPS-stimulated group was taken to be 100%. Band intensities were quantified by densitometry and are indicated as the percent change relative to that of the LPS-stimulated group. Compounds **1**, **2** and dexamethasone (Dex) significantly inhibited LPS-induced iNOS protein expression in macrophages. The experiment was repeated three times (* *p* < 0.05, significantly different from the LPS-stimulated group).

In the* in vitro* anti-inflammatory activity test, the upregulation of the pro-inflammatory iNOS (inducible nitric oxide synthase) and COX-2 (cyclooxygenase-2) protein expression of LPS (lipopolysaccharide)-stimulated RAW264.7 macrophage cells was evaluated using immunoblot analysis. At a concentration of 10 μM, Compounds **1** and **2** were found to significantly reduce the levels of iNOS to 23.67% ± 1.86% and 31.71% ± 8.75%, respectively, relative to the control cells stimulated with LPS only (Figure 3). Thus, Compounds** 1** and **2 **might be promising as anti-inflammatory agents, as they do not exhibit cytotoxicity to RAW264.7 macrophage cells.

## 3. Experimental Section

### 3.1. General Experimental Procedures

Optical rotation values were measured with a Jasco P-1010 digital polarimeter (Japan Spectroscopic Corporation, Tokyo, Japan). IR spectra were obtained on a Varian Digilab FTS 1000 FT-IR spectrophotometer (Varian Inc., Palo Alto, CA, USA); peaks are reported in cm^−^^1^. NMR spectra were recorded on a Varian Mercury Plus 400 NMR spectrometer (Varian Inc., Palo Alto, CA, USA) using the residual CHCl_3_ signal (δ_H_ 7.26 ppm) as the internal standard for ^1^H NMR and CDCl_3_ (δ_C_ 77.1 ppm) for ^13^C NMR. Coupling constants (*J*) are given in Hz. ESIMS and HRESIMS were recorded using a Bruker 7 Tesla solariX FTMS system (Bruker, Bremen, Germany). Column chromatography was performed on silica gel (230–400 mesh, Merck, Darmstadt, Germany). TLC was carried out on precoated Kieselgel 60 F_254_ (0.25 mm, Merck, Darmstadt, Germany); spots were visualized by spraying with 10% H_2_SO_4_ solution followed by heating. Normal-phase HPLC (NP-HPLC) was performed using a system comprised of a Hitachi L-7110 pump (Hitachi Ltd., Tokyo, Japan), a Hitachi L-7455 photodiode array detector (Hitachi Ltd., Tokyo, Japan) and a Rheodyne 7725 injection port (Rheodyne LLC, Rohnert Park, CA, USA). A semi-preparative normal-phase column (Hibar 250 × 10 mm, LiChrospher Si 60, 5 μm, Merck, Darmstadt, Germany) was used for HPLC. The reverse phase HPLC (NP-HPLC) was performed using a system comprised of a Hitachi L-7100 pump (Hitachi Ltd., Tokyo, Japan), a Hitachi L-2455 photodiode array detector (Hitachi Ltd., Tokyo, Japan), a Rheodyne 7725 injection port (Rheodyne LLC., Rohnert Park, CA, USA) and a Varian Polaris 5 C-18-A column (25 cm × 10 mm, 5 μm).

### 3.2. Animal Material

Specimens of the octocorals *Briareum* sp. were collected by hand using scuba equipment off the coast of southern Taiwan in July, 2011, and stored in a freezer until extraction. A voucher specimen (NMMBA-TW-SC-2011-77) was deposited in the National Museum of Marine Biology and Aquarium. This organism was identified by comparison with previous descriptions [45,46,47,48,49].

### 3.3. Extraction and Isolation

Sliced bodies of *Briareum* sp. (wet weight, 6.32 kg; dry weight, 2.78 kg) were extracted with a mixture of methanol (MeOH) and dichloromethane (DCM) (1:1). The extract was partitioned between ethyl acetate (EtOAc) and H_2_O. The EtOAc layer was separated on silica gel and eluted using *n*-hexane/EtOAc (stepwise, 100:1, pure EtOAc) to yield 18 fractions, A–R. Fractions M, N, O and P were combined and further separated on silica gel and eluted using *n*-hexane/EtOAc (stepwise, 4:1, pure EtOAc) to afford 30 fractions, M1–M30. Fraction M4 was further separated by normal-phase HPLC (NP-HPLC) using a mixture of *n*-hexane and EtOAc as the mobile phase to afford **2** (2:1, 0.7 mg). Fraction Q was chromatographed on silica gel and eluted using *n*-hexane/EtOAc (stepwise, 4:1, pure EtOAc) to afford 20 fractions, Q1–Q20. Fraction Q1 was separated by reverse-phase HPLC (RP-HPLC), using a mixture of acetonitrile and H_2_O as the mobile phase to afford **1** (30:70, 0.7 mg).

Briarenolide K (**1**): white powder; mp 215–216 °C; ${\left[\alpha \right]}_{D}^{27}$ −291 (*c* 0.2, CHCl_3_); IR (neat) ν_max_ 3366, 1781, 1733 cm^−1^; ^1^H (400 MHz, CDCl_3_) and ^13^C (100 MHz, CDCl_3_) NMR data (see Table 1); ESIMS: *m/z* 561 [M + Na]^+^; HRESIMS: *m/z* 561.19435 (calcd. for C_26_H_34_O_12_ + Na, 561.19425).

Briarenolide L (**2**): white powder; mp 110–111 °C; ${\left[\alpha \right]}_{D}^{27}$ −177 (*c* 0.2, CHCl_3_); IR (neat) ν_max_ 3461, 1780, 1739 cm^−1^; ^1^H (400 MHz, CDCl_3_) and ^13^C (100 MHz, CDCl_3_) NMR data (see Table 2); ESIMS: *m/z* 601 [M + Na]^+^; HRESIMS: *m/z* 601.26221 (calcd. for C_30_H_42_O_11_ + Na, 601.26193).

### 3.4. In Vitro Anti-Inflammatory Assay

The murine macrophage (RAW264.7) cell line was purchased from ATCC. The* in vitro* anti-inflammatory activity of Compounds **1** and **2** was measured by examining the inhibition of lipopolysaccharide (LPS)-induced upregulation of pro-inflammatory iNOS (inducible nitric oxide synthase) and COX-2 (cyclooxygenase-2) protein expression in macrophage cells using Western blotting analysis [52,53,54]. Briefly, inflammation in macrophages was induced by incubating them for 16 h in a medium containing only LPS (10 ng/mL) without compounds. For the anti-inflammatory activity assay, Compounds **1**, **2** and dexamethasone (10 μM) were added to the cells 10 min before the LPS challenge. The cells were then for western blot analysis. The immunoreactivity data were calculated with respect to the average optical density of the corresponding LPS-stimulated group. For statistical analysis, the data were analyzed by a one-way analysis of variance (ANOVA), followed by the Student–Newman–Keuls *post hoc* test for multiple comparisons. A significant difference was defined as a *p*-value of <0.05.

## 4. Conclusions

Our continuing investigations demonstrated that the octocoral, *Briareum* sp., is a good source of bioactive substances. Compounds **1** and **2** are potentially anti-inflammatory and may become lead compounds in future marine anti-inflammation drug development [55,56]. These results suggest that continuing investigation of novel secondary metabolites together with the potentially useful bioactivities from this marine organism are worthwhile for future drug development. The octocoral *Briareum* sp. will be transplanted to culturing tanks located in the National Museum of Marine Biology and Aquarium, Taiwan, for extraction of additional natural products to establish a stable supply of bioactive material.

## Supplementary Files

Supplementary File 1
