# Supplementary material for: Briarenolides K and L, New Anti-Inflammatory Briarane Diterpenoids from an Octocoral Briareum sp. (Briareidae)
Source: Mar Drugs. 2015 Feb 13;13(2):1037–50. doi: 10.3390/md13021037 (PMC4344617; doi:10.3390/md13021037)
Supplement: Supplementary File 1 [file marinedrugs-13-01037-s001.pdf]

Supplementary Information

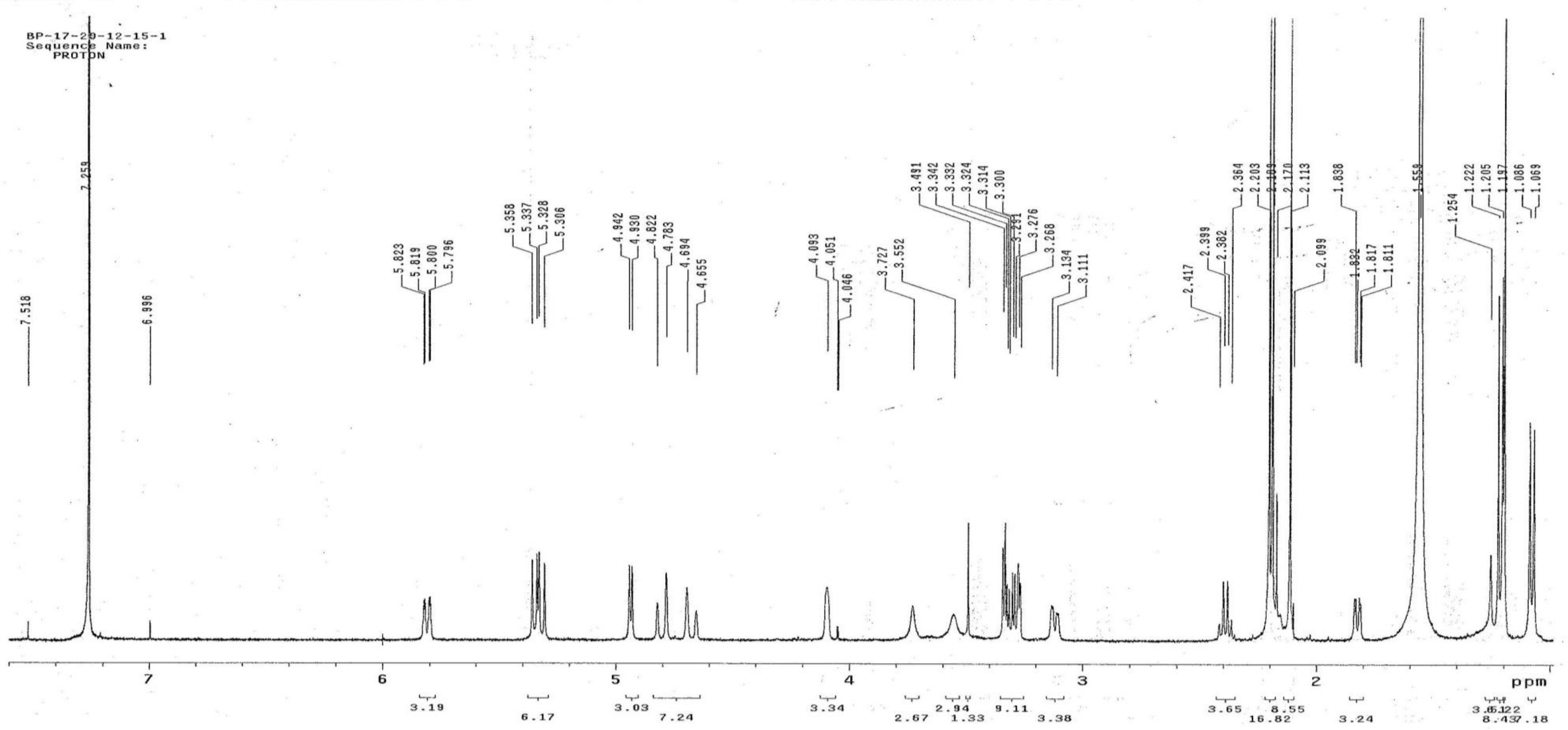

Figure S1. ^{1}H NMR spectrum (400 MHz) of compound **1** in $CDCl_3$.

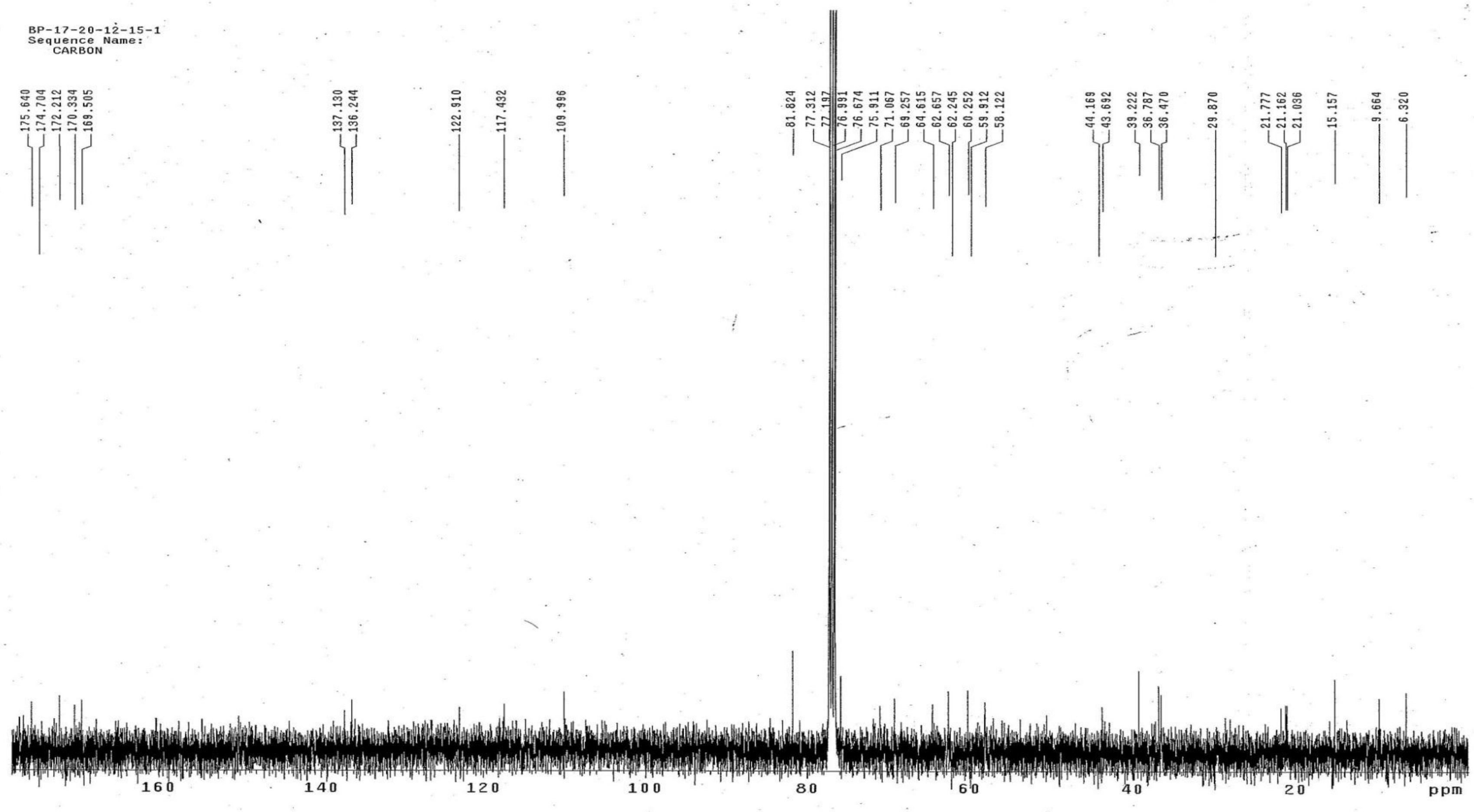

Figure S2. ^{13}C NMR spectrum (100 MHz) of compound **1** in $CDCl_3$.

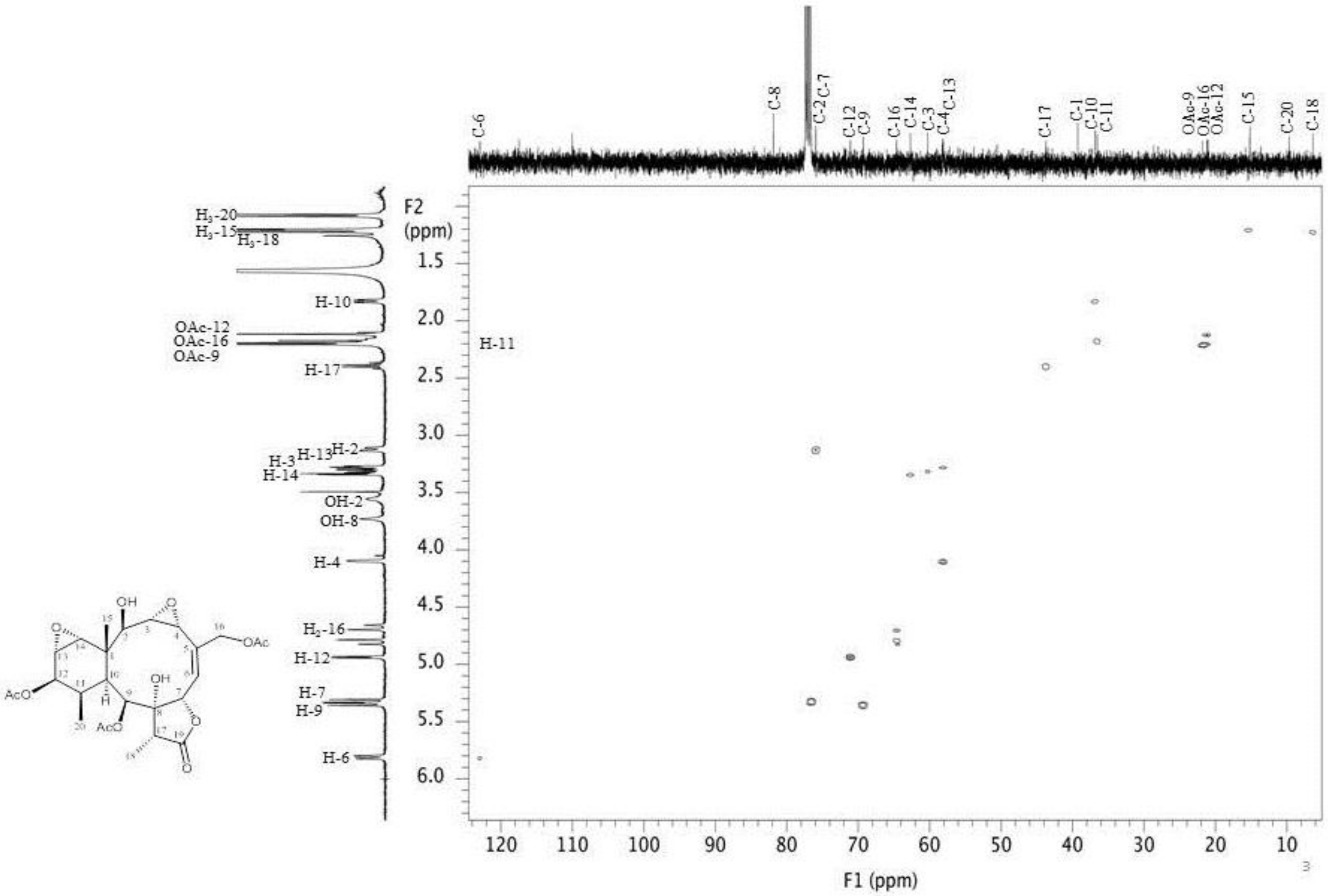

Figure S3. gHSQC spectrum (400 MHz) of compound **1** in $CDCl_3$.

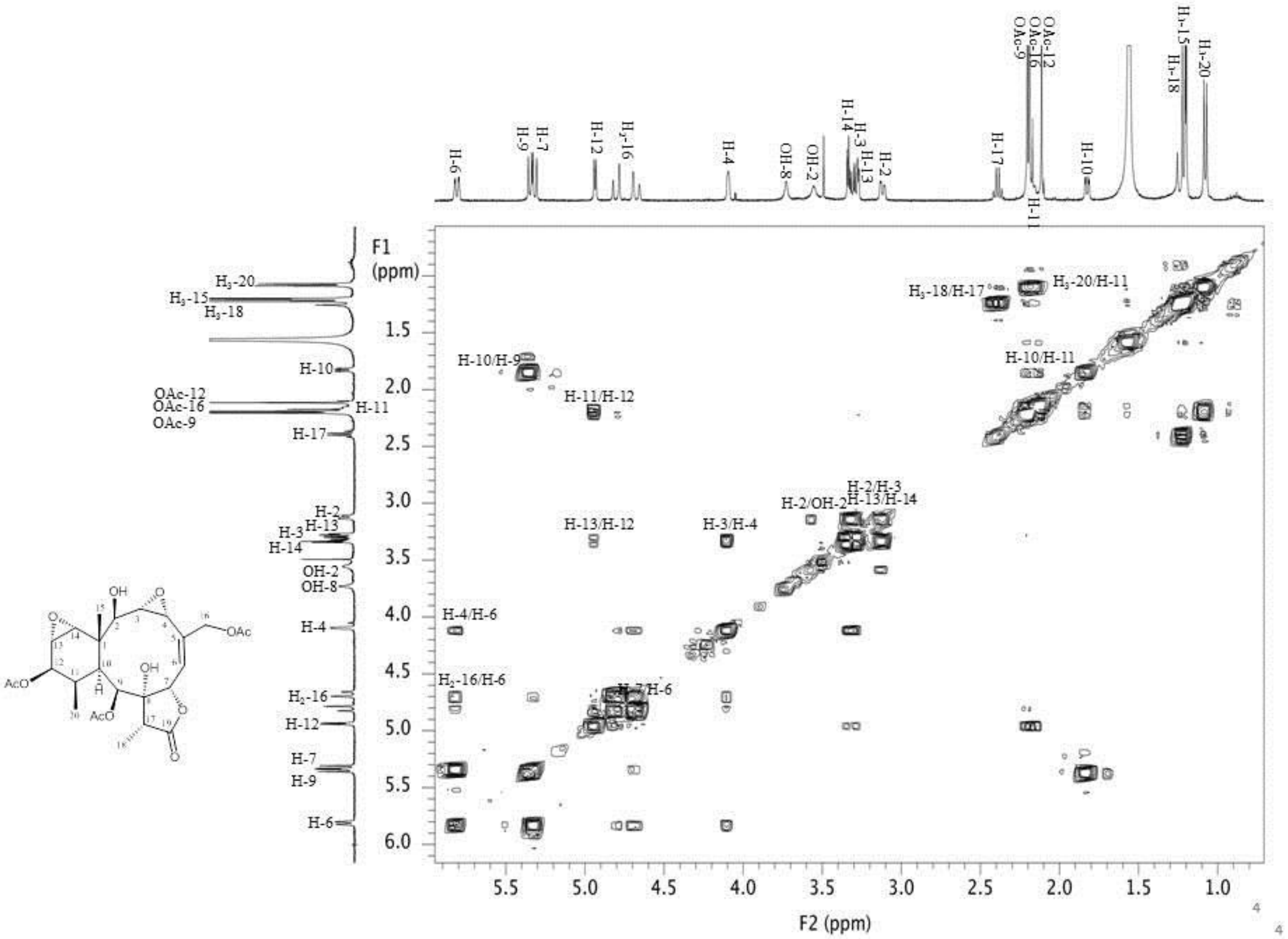

Figure S4. COSY spectrum (400 MHz) of compound **1** in $CDCl_3$.

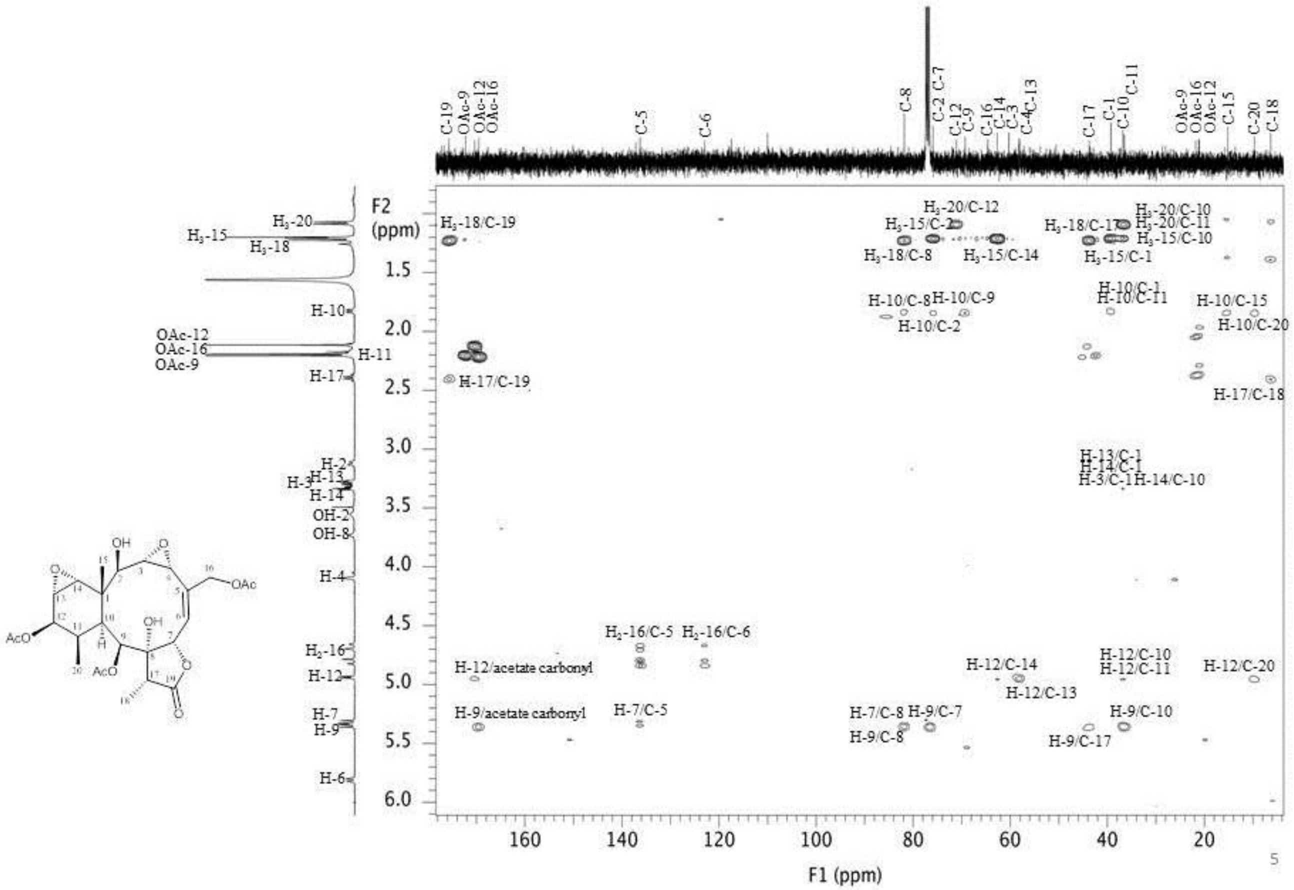

Figure S5. gHMBC spectrum (400 MHz) of compound **1** in $CDCl_3$.

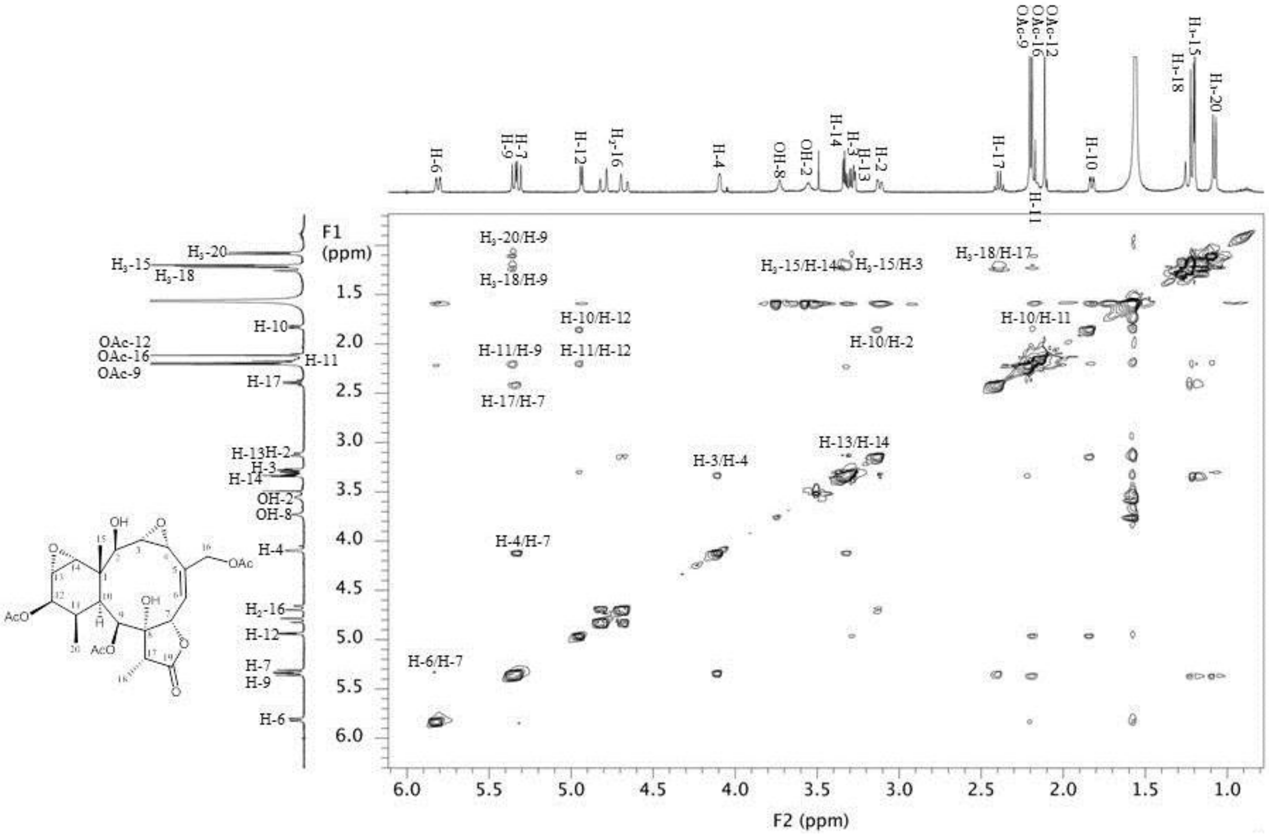

Figure S6. NOESY spectrum (400 MHz) of compound **1** in $CDCl_3$.

Mass Spectrum SmartFormula Report

Analysis Info

Analysis Name	D:\Data\a2\bp172012151_000007.d	5/29/2014 3:00:05 PM
Method	20140527	
Sample Name	BP-17-20-12-15-1	Instrument: FT-MS solariX
Comment	ESI Positive	

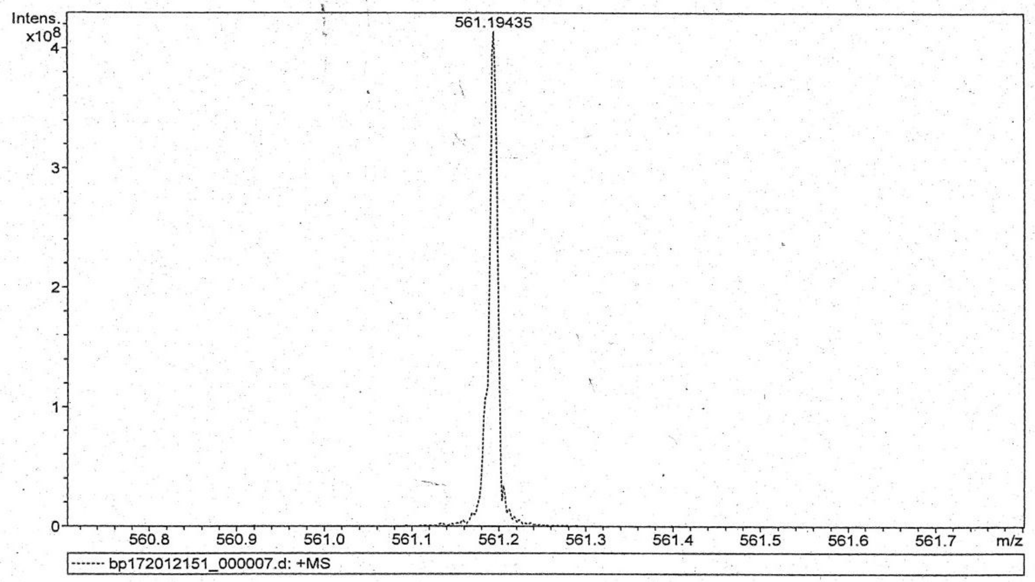

Meas. m/z	#	Formula	Score	m/z	err [mDa]	err [ppm]	mSigma	rdb	e⁻ Conf	N-Rule
561.19435	1	C 26 H 34 Na O 12	100.00	561.19425	-0.10	-0.18	19.4	9.5	even	ok

Figure S7. HRMS of compound **1**.

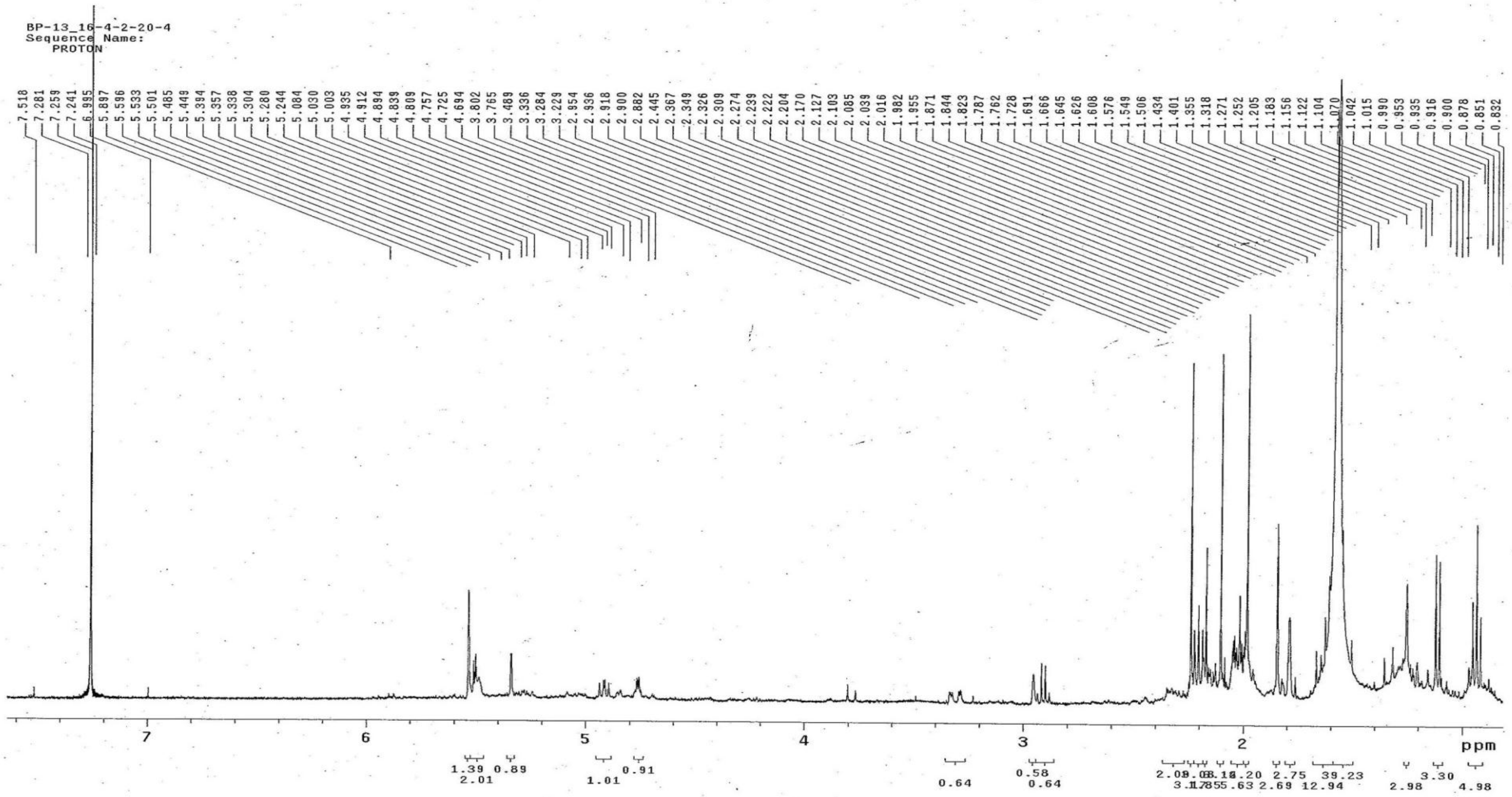

Figure S8. ^{1}H NMR spectrum (400 MHz) of compound **2** in $CDCl_3$.

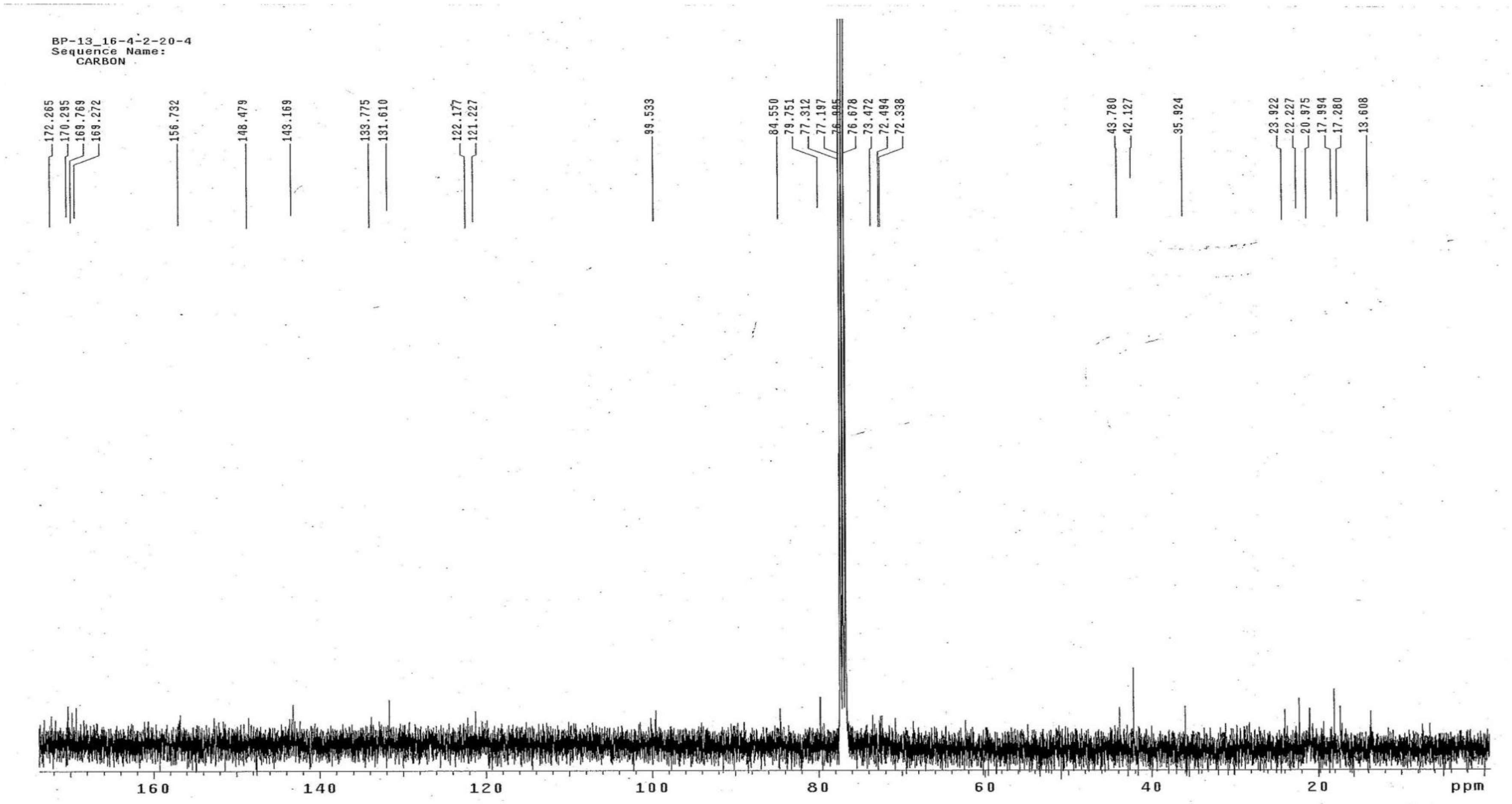

Figure S9. ^{13}C NMR spectrum (100 MHz) of compound **2** in $CDCl_3$.

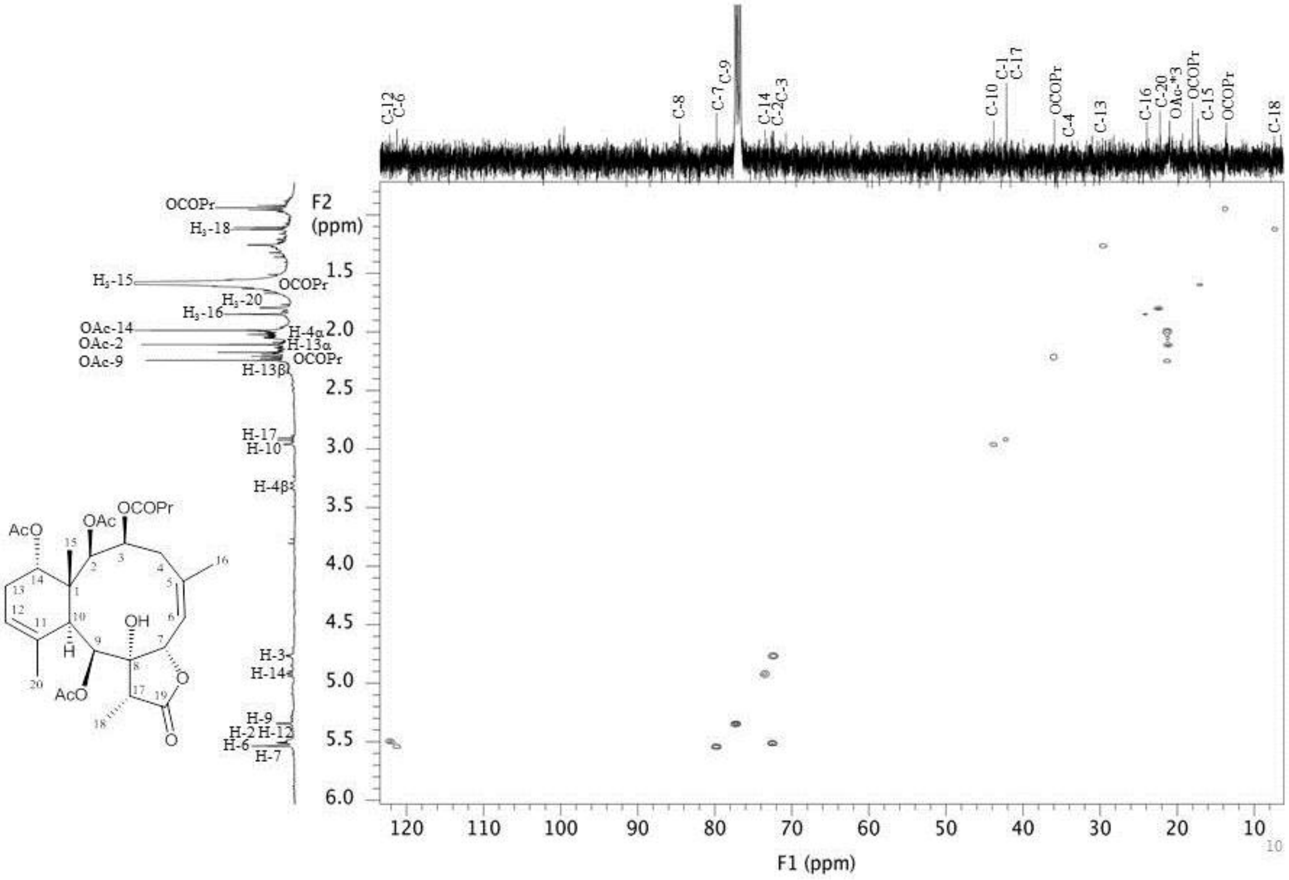

Figure S10. gHSQC spectrum (400 MHz) of compound **2** in $CDCl_3$.

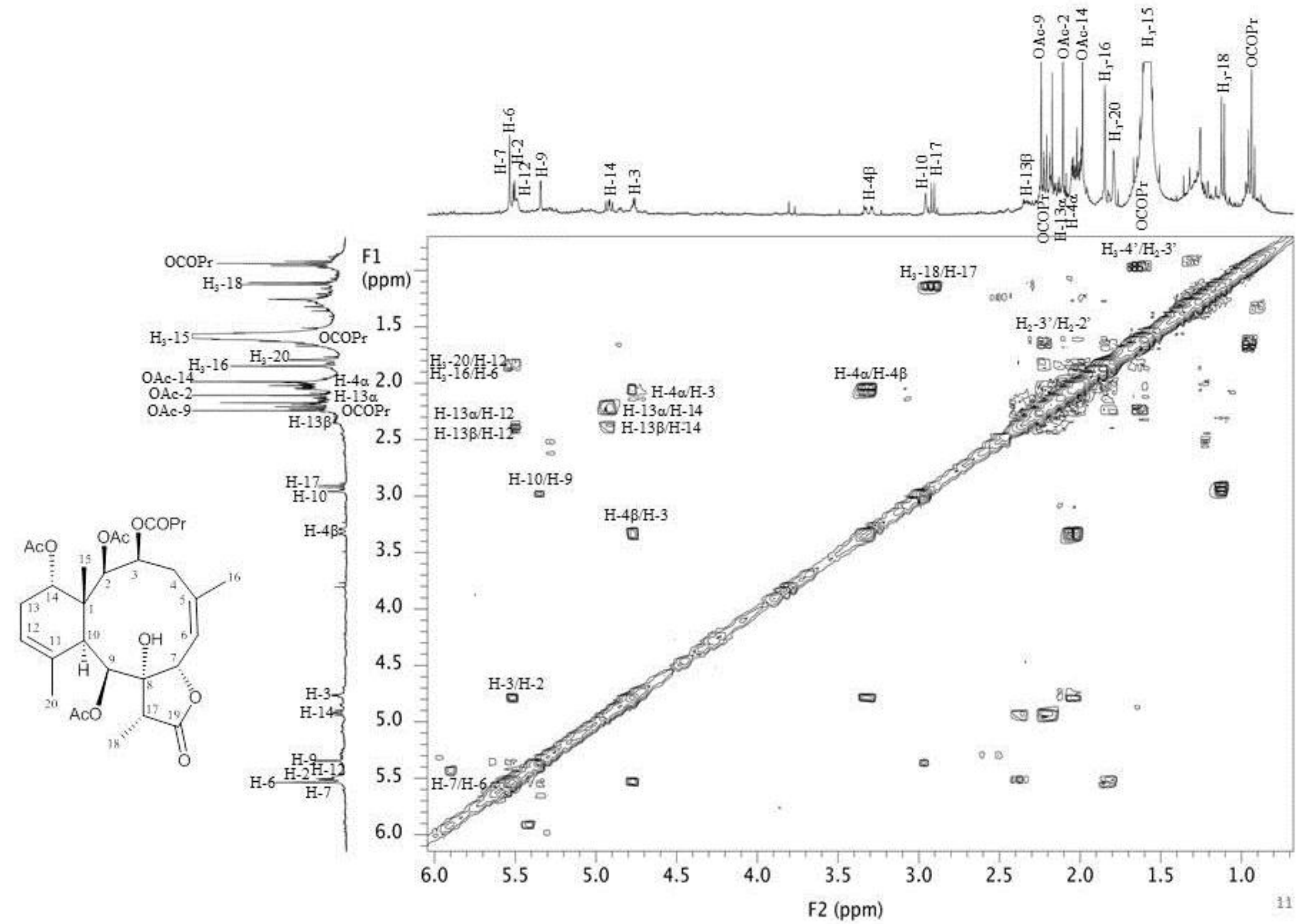

Figure S11. COSY spectrum (400 MHz) of compound **2** in $CDCl_3$.

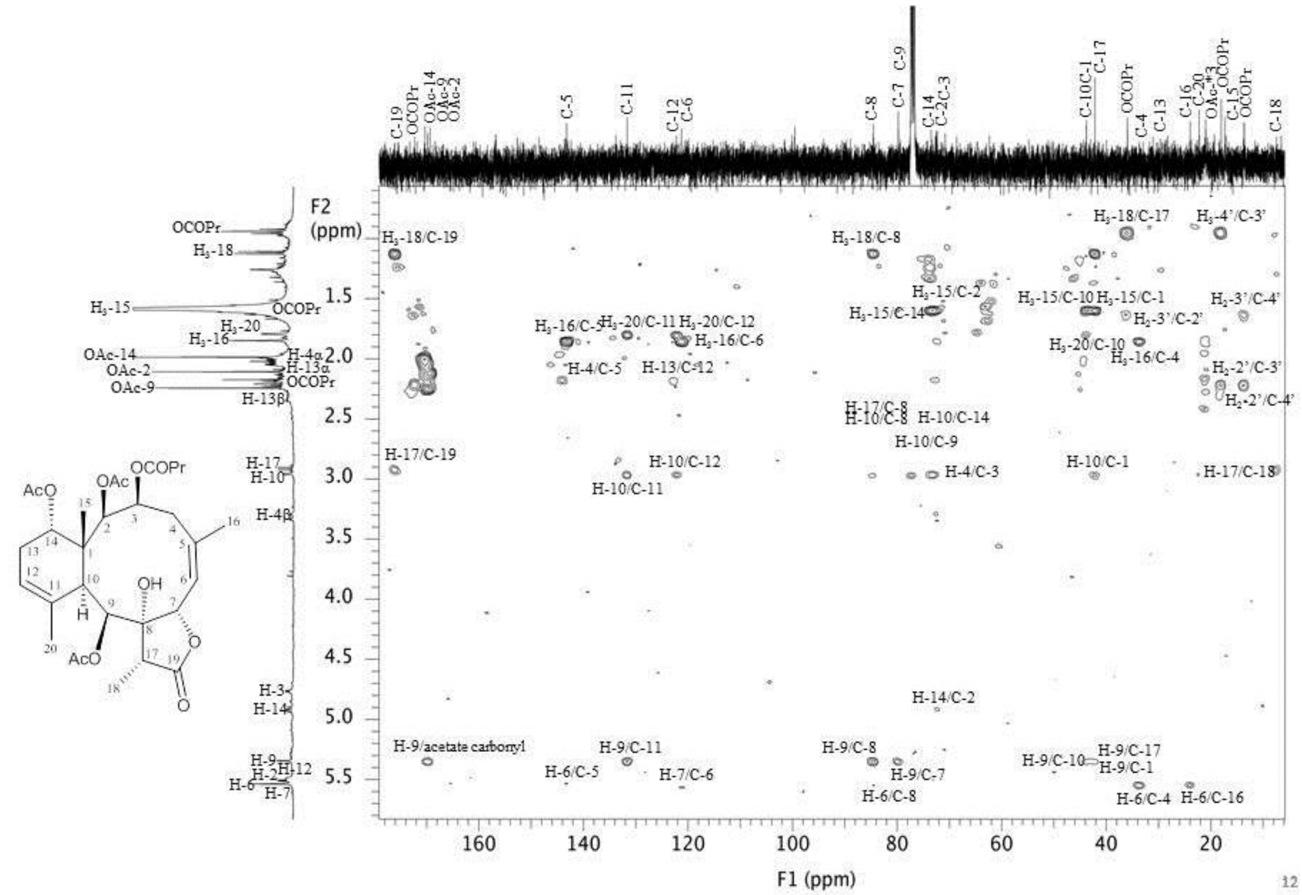

Figure S12. gHMBC spectrum (400 MHz) of compound **2** in $CDCl_3$.

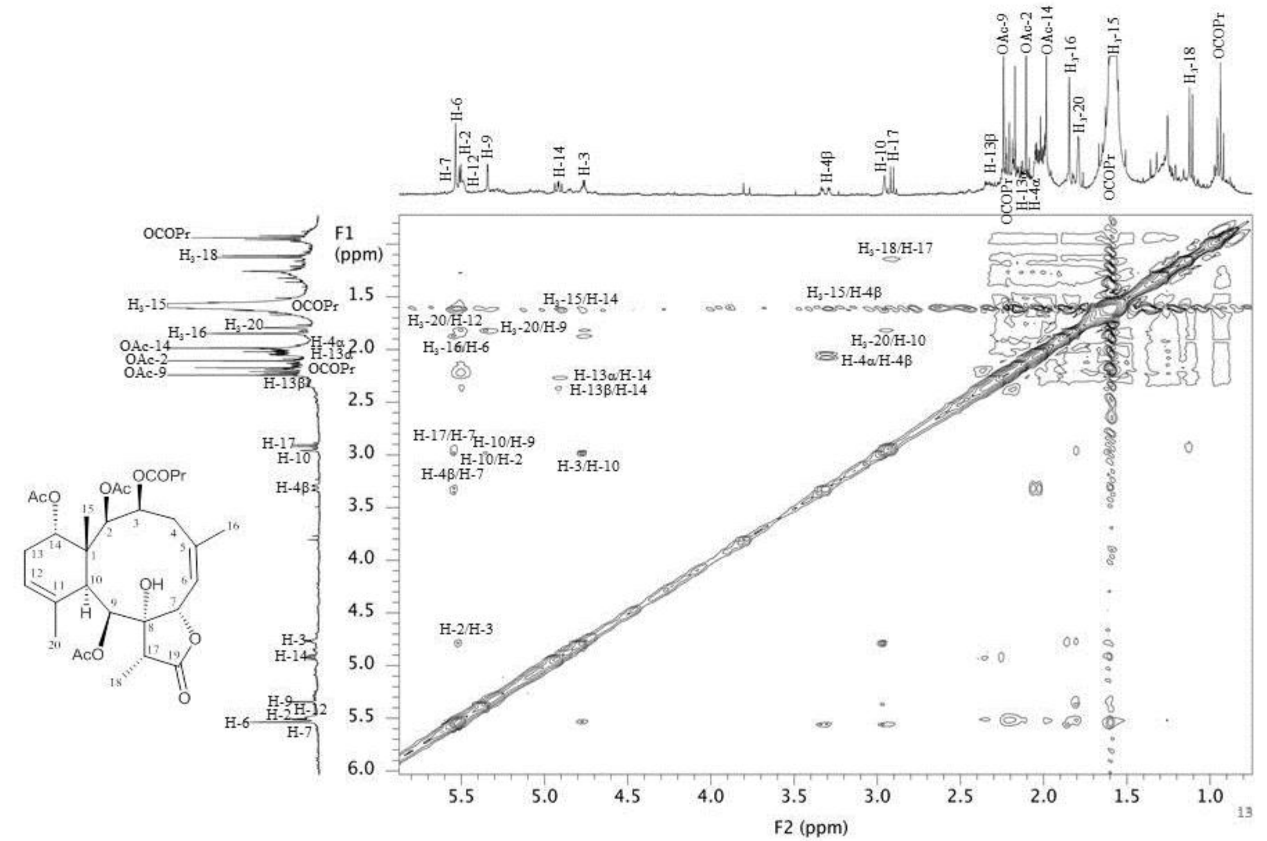

Figure S13. NOESY spectrum (400 MHz) of compound **2** in $CDCl_3$.

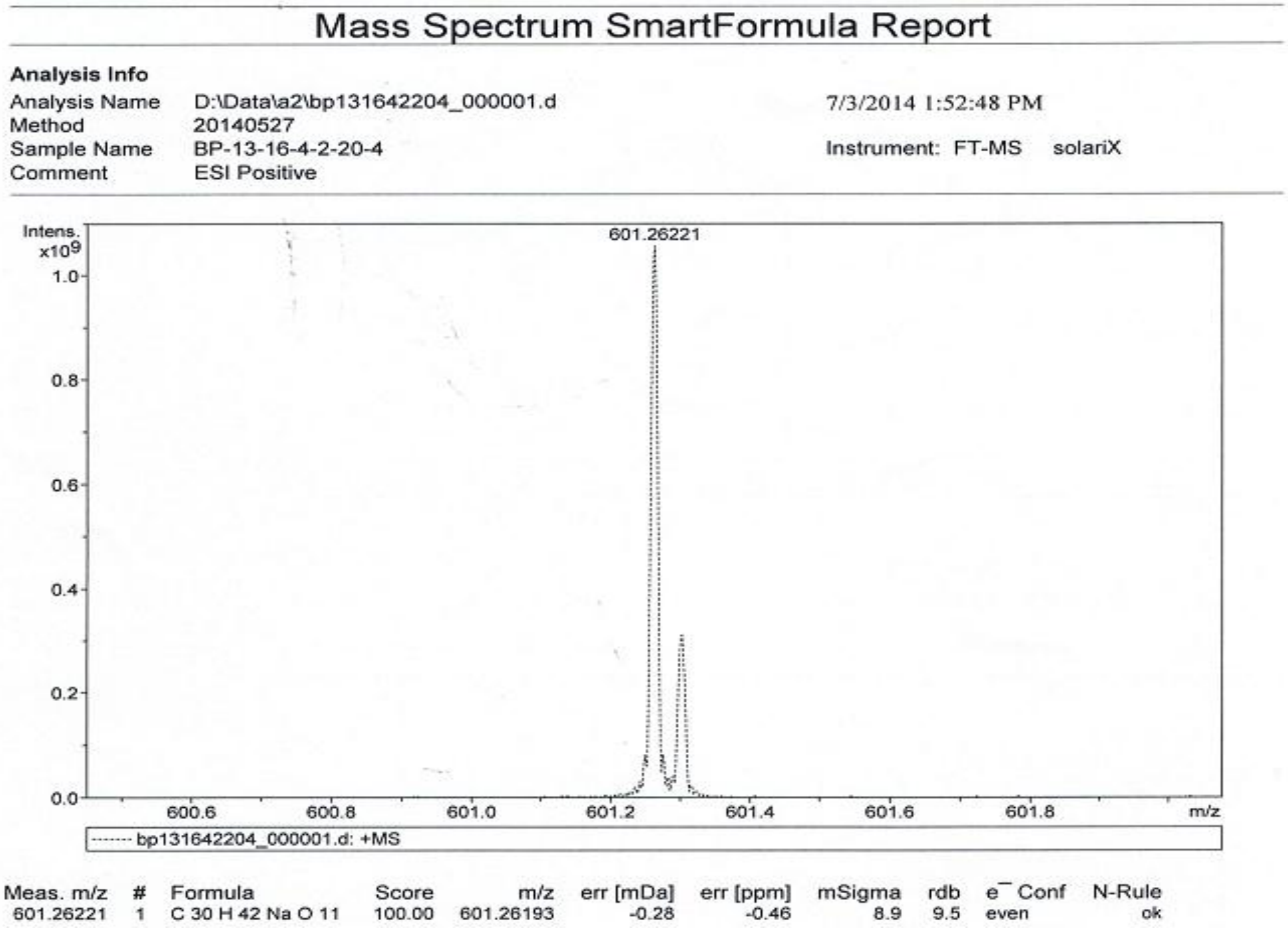

Mass Spectrum SmartFormula Report

Analysis Info

Analysis Name	D:\Data\a2\bp131642204_000001.d	7/3/2014 1:52:48 PM
Method	20140527	
Sample Name	BP-13-16-4-2-20-4	Instrument: FT-MS solariX
Comment	ESI Positive	

Meas. m/z	#	Formula	Score	m/z	err [mDa]	err [ppm]	mSigma	rdb	e⁻ Conf	N-Rule
601.26221	1	C 30 H 42 Na O 11	100.00	601.26193	-0.28	-0.46	8.9	9.5	even	ok

Figure S14. HRMS of compound **2**.

© 2015 by the authors; licensee MDPI, Basel, Switzerland. This article is an open access article distributed under the terms and conditions of the Creative Commons Attribution license (http://creativecommons.org/licenses/by/4.0/).
